# CXCL14 promotes osteosarcoma cell migration and invasion by inducing epithelial-to-mesenchymal transition

**DOI:** 10.7150/ijms.127140

**Published:** 2026-05-11

**Authors:** Chun-Han Hou, Ming-Lung Hsu, Chen-Hsuan Wang, Ju-Fang Liu, Po-Chun Chen

**Affiliations:** 1 Department of Orthopedic Surgery, National Taiwan University Hospital, Taipei 100229, Taiwan.; 2 Department of Life Science, National Taiwan Normal University, Taipei 11677, Taiwan.; 3 School of Oral Hygiene, College of Oral Medicine, Taipei Medical University, Taipei 10031, Taiwan.; 4 Department of Medical Research, China Medical University Hospital, China Medical University, Taichung 404328, Taiwan.

**Keywords:** CXCL14, osteosarcoma, EMT, AKT, MAPK, NF-κB

## Abstract

Osteosarcoma, an aggressive malignancy of long bones in children and young adults, responds poorly to conventional chemotherapy, and distant metastasis remains the principal cause of death. Here we identify chemokine CXCL14 as a driver of epithelial-to-mesenchymal transition (EMT) and metastatic behavior. CXCL14 expression was markedly higher in osteosarcoma cells than in normal osteoblasts. Knockdown of CXCL14 significantly reduced cell migration and wound closure, accompanied by a shift from spindle-like to cobblestone morphology and restoration of epithelial markers. Recombinant CXCL14 promoted EMT with dose-dependent effects. Western blotting showed CXCL14 suppressed E-cadherin while elevating N-cadherin and vimentin, consistent with EMT induction. Pharmacologic and genetic inhibition revealed that PI3K/AKT, MAPK, and NF-κB signaling cascades mediate CXCL14-driven EMT. In an orthotopic mouse model, silencing CXCL14 retarded tumor growth and reduced mesenchymal marker expression *in vivo*. Collectively, our results demonstrate that CXCL14 activates PI3K/AKT/MAPK/NF-κB pathways to promote EMT and osteosarcoma progression. Therapeutic targeting of CXCL14 or its downstream signaling may suppress metastasis and improve outcomes for patients with osteosarcoma. These data position CXCL14 as both a biomarker of aggressive phenotypes and an actionable molecular target in pediatric bone cancer patients, warranting preclinical and clinical validation.

## Introduction

Osteosarcoma is the most prevalent and aggressive primary bone tumor, predominantly impacting adolescents and children, with an incidence of approximately 1-3 cases per million people 1. Osteosarcoma is highly aggressive, with a metastasis rate of approximately 20%, most commonly spreading to the lungs and other bones. Despite ongoing research, the mechanisms through which osteosarcoma develops remain unclear, although it is most likely derived from mesenchymal stem cells or osteoprogenitor cells 2. Clinically, osteosarcoma is usually treated using chemotherapy or radiation; however, due to the high tumor recurrence and metastasis rates to other organs, patient prognoses remain poor, with a five-year survival rate under 20%. Subsequently, most patients eventually undergo amputation surgery, making the treatment of this cancer a critical issue 3.

Similar to other solid tumors, osteosarcoma has a strong tendency to metastasize to the lungs; the lungs form the most common site for metastasis in clinical cases 4. The possible molecular mechanisms through which metastasis occurs involve triggering tumor cell migration and invasion, allowing cells to pass through the extracellular matrix into the bloodstream, resisting apoptosis, and, ultimately, promoting osteosarcoma metastasis by establishing cell adhesion, which enables metastatic cells to attach to a new environment 5. Epithelial-mesenchymal transition (EMT) is essential for vertebrate development and tissue maintenance and has recently been linked to disease progression, including tissue fibrosis and cancer 6. Meanwhile, new light has been shed on EMT following extensive research, clarifying the invasive and metastatic behaviors observed during cancer progression 7. EMT is a multifaceted process in which epithelial cells lose their polarity and generate a mesenchymal cell-like property 7, 8. Moreover, there is a reduction in epithelial phenotype markers during EMT, such as the cell adhesion molecule E-cadherin, ZO-1, and claudin, accompanied by an increase in the expression of mesenchymal phenotype markers, including N-cadherin, vimentin, and fibronectin. Transcription factors such as Twist, Snail1, Slug, and the Zeb family, which regulate EMT by suppressing E-cadherin, have been found to be associated with the pathological mechanisms underlying osteosarcoma 9.

Chemokines are signaling molecules secreted by diverse cell types throughout the body, which act by attracting nearby cells through chemotaxis and by regulating homeostasis and inflammatory processes within the local environment 10. The expression of CXCL14 in various tumors is inconsistent. However, some studies suggest that CXCL14 expression increases in epithelial cells, while other literature indicates that tumor-associated fibroblasts and infiltrating lymphocytes are common sources of CXCL14 overexpression 11-13. The role of CXCL14 in tumor progression has been studied in various cancers. Indeed, CXCL14 has been shown to promote cell growth, migration, and chemoresistance in breast cancer 14. Additionally, the release of CXCL14 in the tumor microenvironment by fibroblasts can elevate the levels of mesenchymal markers in cancer cells, trigger EMT, and facilitate lung metastasis 12. These findings indicate that CXCL14 may contribute to tumor promotion under specific circumstances. However, some studies have suggested that *CXCL14* may function as a tumor suppressor gene. In an experimental mouse oral cancer model, transfection-induced increased CXCL14 expression notably reduced tumor growth 15. Beyond oral cancer, *CXCL14* transgenic mice also exhibited inhibited tumor growth in melanoma and colon cancer, attributed to increased natural killer (NK) cell activity 16. Moreover, CXCL14 has been proven to inhibit tumor growth in liver cancer. CXCL14 expression is notably reduced in tissues from patients with hepatitis B virus (HBV)-positive liver cancer, and variations in the *CXCL14* gene are linked to progression due to HBV infection 17. In conclusion, similar to other chemokines, the role of CXCL14 in tumor cells varies depending on the experimental model system and the types of cells through which it is secreted 18. A previous report demonstrated that CXCL14 is highly expressed in osteosarcoma tissues and cells, and this expression is strongly correlated with the prognosis of osteosarcoma patients 19. However, the roles of CXCL14 in osteosarcoma progression remain to be explored.

This study aimed to show that CXCL14 actively drives osteosarcoma progression. Silencing CXCL14 reduces cell migration, invasion, and EMT *in vitro* and tumor growth in an intratibial mouse model. Mechanistic tests have revealed that CXCL14 activates the PI3K/AKT, mitogen-activated protein kinase (MAPK), and NF-κB signaling pathways, which increase the expression of EMT regulators, such as Twist and Snail, thereby promoting cell migratory potential. Our findings demonstrate the signal pathway through which CXCL14 promotes osteosarcoma progression and identify AKT, ERK1/2, and p65 as potential therapeutic targets.

## Materials and Methods

### Materials

Horseradish peroxidase-conjugated anti-mouse (Cat. No. 115-035-174) and anti-rabbit IgG (Cat. No. 211-032-171) were sourced from Jackson ImmunoResearch (West Grove, PA, USA). The primary antibodies targeting E-cadherin (Cat. No. #14472S), N-cadherin (Cat. No. #4061), vimentin (Cat. No. #5741), Snail (Cat. No. #3879), Twist (Cat. No. #69366), p-AKT (Cat. No. #4060S), AKT (Cat. No. #4691S), p-ERK (Cat. No. #9101), ERK (Cat. No. #4695), p-JNK (Cat. No. #4668S), JNK (Cat. No. #9252), p-p38 (Cat. No. #9211S), p38 (Cat. No. #8690S), p-p65 (Cat. No. #3033S), p65 (Cat. No. #8242S), and β-actin (Cat. No. A5441), were purchased from Cell Signaling Technology (Danvers, MA, USA) and Sigma-Aldrich (St. Louis, MO, USA). All other chemicals were sourced from Sigma-Aldrich (St. Louis, MO, USA).

### Cell culture

All osteosarcoma cell lines (HOS, U2OS, and 143B) and the normal osteoblast cell line (hFOB 1.19) were purchased from the Bioresource Collection and Research Center (BCRC; Hsinchu, Taiwan). The cells were cultured using the medium outlined in the official guidelines. Additionally, complete medium was prepared using 10% fetal bovine serum (FBS), 2 mM glutamine, penicillin/streptomycin (Invitrogen, Carlsbad, CA), and Normocin® (InvivoGen, San Diego, CA, USA) supplementation to prevent contamination by mycoplasma, bacteria, and fungi. The cells were then incubated at 37 °C in a 5% CO_2_ incubator.

### Cell viability assay

A resazurin cell viability assay (Biotium; Fremont, CA, USA) was conducted to assess the viability of osteosarcoma cells following CXCL14 treatment. Osteosarcoma cells were plated at a concentration of 20,000 cells per well in 96-well plates and exposed to various concentrations of CXCL14. After the specified exposure times, the media were removed, and 100 µL of fresh media containing 10 µL of resazurin solution was added. The plates were incubated in the dark at 37 °C for an additional hour. Cell viability was assessed by measuring fluorescence emission at 590 nm with an excitation wavelength of 540 nm.

### Bulk RNA-sequencing

Total RNA was extracted by using Easyblue^TM^ total RNA extraction kit (iNtRON Biotechnology, WA, USA). RNA-sequencing analysis were carried out by Genomics Co. Ltd. on the NovaSeq^®^ 6000 (Illumina, CA, USA) platform. The difference expression of genes (DEGs) was analyzed using DESeq2 and then subjected to enrichment analysis of KEGG pathways.

### Bioinformatics analysis

Gene expression data for CXCL14 in osteosarcoma specimens and normal osteoblasts were retrieved from the Gene Expression Omnibus (GEO) database (GSE12865 and GSE42352). The *CXCL14* gene expression profiles were extracted using the GEO data analysis tools. A t-test was conducted to evaluate the differences in CXCL14 expression between normal cells and tumor tissues. A *p*-value of ≤ 0.05 was applied to determine statistical significance.

### Selection of a migration-prone stable cell line

To generate the MG63 osteosarcoma cell line with high migratory ability, referred to as M5 and M10, Transwell inserts were utilized within a 6-well plate featuring an 8 μm pore size. Initially, 100 μL of serum-free medium containing 1 × 10^4^ MG63 cells was introduced into the upper chamber, while 300 μL of growth medium supplemented with 10% FBS was added to the lower chamber. Following a 24-hour incubation period, the cells that migrated through the Transwell membrane to the lower compartment were detached using trypsin and subsequently cultured, resulting in the establishment of the first subclone, designated MG63 (M1). The cells were then maintained in culture for an additional two days before undergoing a second round of selection. This migration selection protocol was systematically repeated for a total of five and ten rounds, leading to the development of the MG63 (M5) and MG63 (M10) subclones, respectively.

### Western blot analysis

Total cell lysates were obtained from various experimental groups, and the proteins were subsequently separated using SDS-PAGE before being transferred to a PVDF membrane. Then, the membrane was blocked with 4% BSA at room temperature for one hour, after which it was incubated with the specified primary antibody (1:1000 dilution) targeting CXCL14, E-cadherin, N-cadherin, vimentin, Snail, Twist, p-ERK, ERK, p-JNK, JNK, p-p38, p38, p-p65, and p65 at room temperature for 1 hour. Following three TBST washes, the membrane was incubated with an anti-rabbit HRP-conjugated secondary antibody (diluted 1:1000) at room temperature for 1 hour. After another three TBST washes, the membrane was developed using an enhanced chemiluminescence substrate and imaged on a CCD-based imaging system, MultiGel-21 (TOP BIO CO., New Taipei City, Taiwan). Finally, quantification of the results was conducted using ImageJ software (National Institutes of Health, USA).

### Quantitative PCR analysis

The extracted RNA was converted to complementary DNA (cDNA) using Magic RT cDNA synthesis kit (Bio-Genesis, ROC). Real-time quantitative polymerase chain reaction (qPCR) analysis was conducted utilizing the Taqman® system (Applied Biosystems, Foster City, CA). Primers and probes (twist, Hs04989912_s1; snail, Hs00195591_m1; E-cadherin, Hs01023895_m1; N-cadherin, Hs00983056_m1; vimentin, Hs00958111_m1) used for qPCR were purchased from Applied Biosystems (CA, USA). Each 25 µL reaction comprised KAPA PROBE FAST qPCR Master Mix (Sigma-Aldrich), 100 ng of cDNA, specific primers, and a Taqman^®^ probe. All primers and probes targeting the genes of interest were procured from Applied Biosystems (CA). The real-time qPCR analysis was executed in triplicate using the StepOnePlus Real-Time PCR System (Applied Biosystems, CA). The thermal cycling protocol included an initial polymerase activation step at 95 °C for 10 minutes, followed by 40 cycles of denaturation at 95 °C for 15 seconds and annealing and extension at 60 °C for 60 seconds. The threshold for detection was established above the background level of the non-template control and within the linear amplification range of the target gene, allowing for the calculation of the cycle threshold (_Ct_) at which the transcription template was identified.

### Cell morphology

To investigate EMT modifications in cell culture, we performed a cell scatter assay using osteosarcoma cells. The cells were initially plated in a 24-well plate at a density of 0.5 × 10^4^ cells per well, resulting in the formation of dense colonies within 48 hours. Subsequently, the cells were fixed with 4% paraformaldehyde (PFA) and imaged using a camera mounted on a microscope.

### Wound healing assay

In the wound healing assay, osteosarcoma cells (2 × 10^5^ cells) were cultured in a 6-well plate until an approximate 90% confluency was achieved. Culture-Inserts (ibidi, Gräfelfing, Germany) were employed to create cell-free gaps. Subsequently, the cells were washed with phosphate-buffered saline (PBS) to eliminate any detached cells, after which the cells were subjected to various treatments as specified in the figure legends. Following a 24-hour incubation period, the migration of cells across the gaps was documented using microscopy. The resulting images were then analyzed in comparison to the initial scratch (0 hours) to quantify the number of cells that had migrated into the gaps.

### Migration assay

All cell migration assays were performed utilizing Transwell inserts with a pore size of 8 μm within 24-well plates (Costar, NY). The treated osteosarcoma cells (1 × 10^4^ cells in 200 μL of medium) were introduced into the upper chamber of the Transwell plates, while 300 μL of the same medium was placed in the lower chamber. Following a 24-hour incubation period, the cells on the Transwell inserts were fixed using 4% paraformaldehyde (PFA) and subsequently stained with 0.05% crystal violet. Non-migratory cells retained within the Transwell inserts were removed using Q-tips. Ultimately, the cells that had migrated through the Transwell membranes were photographed via microscopy. Each experimental trial was conducted in at least triplicate.

### Luciferase reporter assay

The cells were cultured in 24-well plates until an approximate confluency of 80% was achieved. The following day, transfection was performed using 0.8 µg of luciferase reporter plasmid in conjunction with Lipofectamine 2000 (LF2000; Invitrogen). Post-transfection, the cells were subjected to various treatment conditions for an additional 24 hours. For lysate preparation, 100 µL of lysis buffer (Promega) was added to each well. Following centrifugation at 13,000 rpm for 2 minutes, the supernatant was collected. The resulting cell lysates were then aliquoted into a black 96-well microplate at a volume of 20 µL per well. An equal volume of luciferase substrate was subsequently added to each sample, and luminescence was quantified using a microplate luminometer. To ensure consistency across samples, the luciferase signal was normalized against the total protein concentration.

### Immunofluorescence staining

Cells were cultured on chamber slides for immunofluorescence staining. Following treatment, as outlined in the figure legends, the cells were washed with PBS and fixed in 3.7% formaldehyde for 10 minutes at ambient temperature. After three additional washes with PBS, the cells were blocked with 4% bovine serum albumin (BSA) for 15 minutes. Subsequently, the cells were incubated with a primary antibody against p65 (dilution 1:100) at room temperature for one hour, followed by another round of washing. The cells were then incubated with a fluorescein isothiocyanate (FITC)-conjugated secondary antibody (dilution 1:100) for an additional hour. Finally, the cells were washed, mounted in a solution containing DAPI, and imaged using a Nikon Ti2 microscopy system (Nikon).

### Generation of stable CXCL14 knockdown cell lines

The CXCL14 and control shRNA lentiviral constructs (pLKO.1) were obtained from the National RNAi Core Facility at Academia Sinica in Taipei, Taiwan. HEK293T cells were employed for the production of the lentivirus. Specifically, the shRNA plasmid was co-transfected into HEK293T cells alongside the packaging vectors pCMV and pMDG. The cell culture supernatants were harvested post-transfection at 24 and 48 hours and subsequently stored at -80 °C. To create stable knockdown clones, HOS and 143B cells were transduced with the collected supernatants in the presence of 8 μg/mL polybrene (Sigma-Aldrich). Following a 48-hour transduction period, cells expressing the shRNA vectors were selected using culture medium supplemented with 10 μg/mL puromycin. Stable clones expressing shRNA were established after a two-week selection period using puromycin.

### Orthotopic xenograft animal model

All *in vivo* experiments were conducted in compliance with the Guidelines for Animal Care established by the Institutional Animal Care and Use Committee at National Taiwan University College of Medicine and College of Public Health in Taipei, Taiwan. (IACUC No. 20210318) The mice utilized in this study were procured from Lasco, also located in Taipei, Taiwan. Male CB17-SCID mice, aged five weeks, received intratibial injections of osteosarcoma cells (2 × 10^6^ cells in 100 μL). A total of five mice were included in each group (control and CXCL14-shRNA). The mice were euthanized six weeks post-implantation of the tumor cells, and the tumors were harvested for subsequent analysis. One mouse in the CXCL14-shRNA group exhibited complete tumor regression; thus, no tumor tissue was available at the time of sacrifice. Therefore, data from the remaining four mice in the CXCL14 shRNA group were used for analysis.

### Immunohistochemistry

Following euthanasia, the tumor tissues were harvested and preserved in a 10% formalin solution. After fixation, the tissues underwent dehydration utilizing a xylene and ethanol mixture. Subsequently, the samples were embedded in paraffin blocks and sectioned into slices of 5 μm thickness. These sections were incubated with primary antibodies, diluted 1:100, at room temperature for 1 hour. Signal detection was performed using the NovoLink Polymer Detection Systems kit (Leica Biosystems, Wetzlar, Germany), according to the manufacturer's guidelines. The staining outcomes were documented through imaging of three randomly selected fields for each analyzed slide.

### Statistical analysis

All values are expressed as the mean ± standard deviation derived from multiple independent experiments. Statistical comparisons between two samples were performed utilizing Student's t-test. Meanwhile, one-way analysis of variance (ANOVA) followed by Fisher's least significant difference (LSD) post hoc test was employed for comparisons involving more than two groups. A *p*-value of less than 0.05 was deemed statistically significant.

## Results

### CXCL14 is highly expressed in osteosarcoma cells and patient specimens

**Figure 1:**
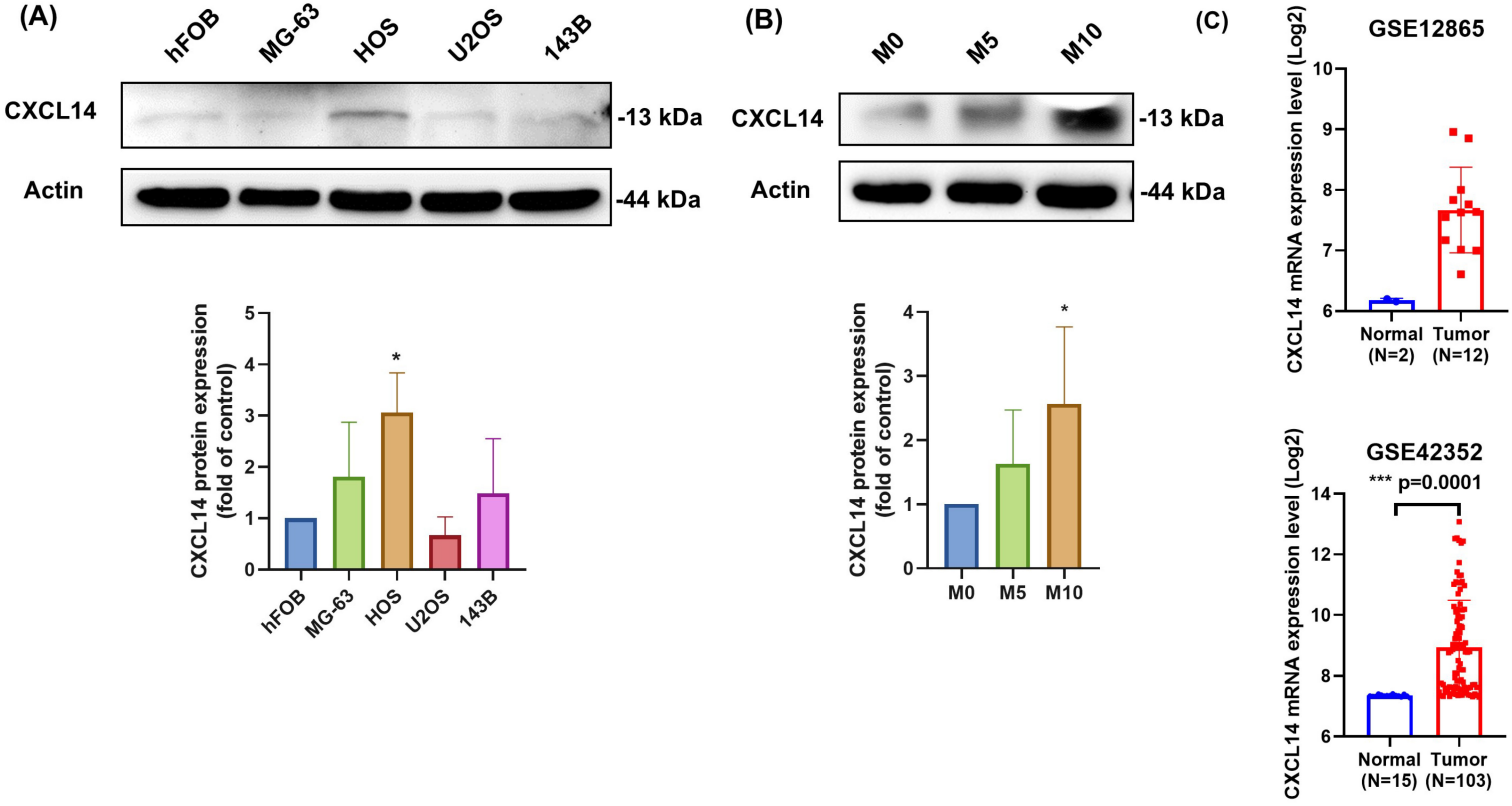
** CXCL14 expression levels in osteosarcoma cell lines and tumor specimens.** (A) Total protein was extracted from human normal osteoblasts (hFOB) and various osteosarcoma cell lines (MG-63, HOS, U2OS, and 143B), and CXCL14 protein expression was assessed using Western blot analysis. The lower panel shows the Western blot quantification results. (B) CXCL14 protein expression was analyzed in MG63 cells with different migratory capacities (M0, M5, and M10) by Western blot. The lower panel presents the quantification results. (C) CXCL14 expression levels in osteosarcoma tissues and normal osteoblasts were retrieved and analyzed from GEO datasets (GSE12865 and GSE42352). Results are presented as the mean ± SEM from at least three independent experiments (N=3-6). *p < 0.05 compared to hFOB, M0, and normal groups.

We first evaluated the CXCL14 levels in human osteoblasts (hFOB) and various osteosarcoma cell lines (MG-63, HOS, U2OS, and 143B), as reported previously 20, 21. We found that CXCL14 expression was upregulated in osteosarcoma cell lines compared to osteoblasts (Fig. 1A). Additionally, we developed an MG63 osteosarcoma cell line with varying levels of migratory ability, including 5 and 10 generations, which reflected the degree of cell malignancy based on their migratory potential. The CXCL14 expression level was also positively related with cell malignancy, with the MG63 M10 cells exhibiting high expression levels (Fig. 1B). We also analyzed the Cancer Genome Atlas (TCGA) dataset for osteosarcoma patients, which showed that CXCL14 was highly expressed in osteosarcoma tissue compared with normal cells (Fig. 1C). Therefore, these results suggest that the expression of CXCL14 is associated with osteosarcoma progression.

### CXCL14 expression positively correlates with migratory potential in osteosarcoma cells

**Figure 2:**
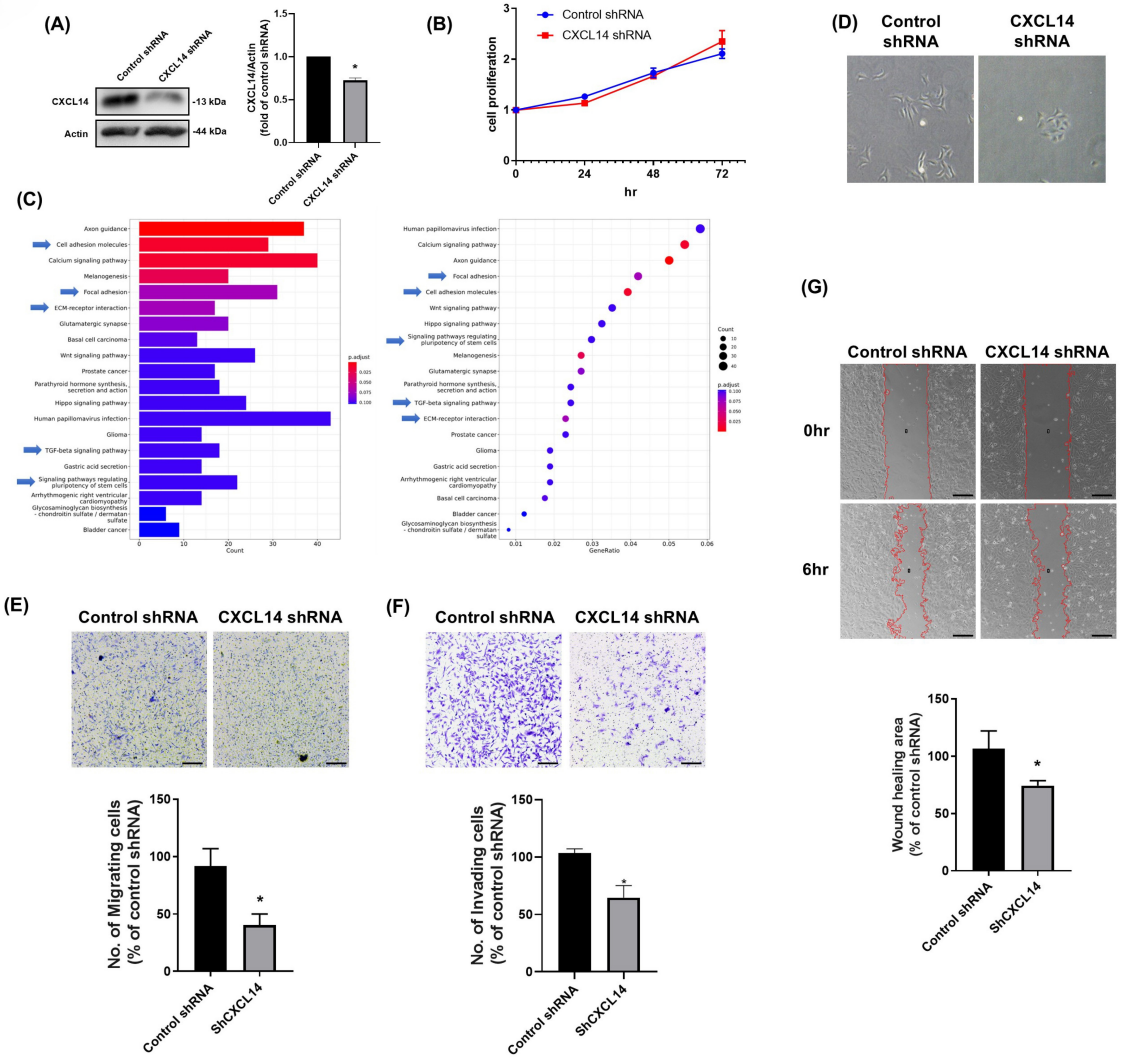
** CXCL14 is involved in the regulation of the migratory ability of osteosarcoma cells.** (A and B) CXCL14 knockdown cells were generated from the HOS cell line, and the protein expression of CXCL14 and cell proliferation were assessed using Western blot and cell viability assays, respectively. (C) The *p*-values and names of the most significantly represented KEGG pathways were determined based on the differentially expressed genes between *CXCL14* shRNA and control shRNA stable clones in HOS cells. (D) *CXCL14* shRNA and control shRNA stable clones in HOS cells were seeded for 24 hours, and cell morphology was captured in photographs. (E-G) The cell migration ability and invasive potential of HOS cells stably expressing CXCL14 shRNA constructs were evaluated using Transwell migration, invasion, and wound healing assays. Results are presented as the mean ± SEM from at least three independent experiments (N=3-6). *p < 0.05 compared to the control shRNA groups. Scale bar= 100 μm.

Next, we generated HOS osteosarcoma cells that stably expressed either control or CXCL14 shRNA and subsequently confirmed the CXCL14 expression levels in these cells (Fig. 2A). CXCL14 knockdown did not alter the rate of cell proliferation (Fig. 2B). We further explored the role of CXCL14 on osteosarcoma progression by evaluating gene expression between CXCL14 shRNA and control shRNA cells. A list of over-represented KEGG pathways was generated using RNA-sequencing (RNA-seq) and bioinformatics analyses (Fig. 2C). The differentially expressed genes (DEGs) were primarily related to cell migratory potential, including cell adhesion and ECM-receptor interaction. Furthermore, the pathways related to the EMT phenotype (TGF-β signaling pathway and signaling pathways regulating stem cell pluripotency) were also highly correlated in the CXCL14 knockdown cells, suggesting that CXCL14 expression was associated with cell mobility and EMT. Therefore, the cell morphology was monitored, and the results indicated that CXCL14 knockdown dramatically changed from a spindle-like to a cobblestone morphology, indicative of a reversal of EMT (Fig. 2D). The migratory and invasive potential of CXCL14 knockdown cells was also confirmed through migration, invasion, and wound healing assays. These data revealed that CXCL14 knockdown reduced cellular migratory and invasive potential (Fig. 2E-2G). These results indicate that CXCL14 enhances cell mobility in osteosarcoma cells.

### CXCL14 promotes EMT in osteosarcoma cells

**Figure 3:**
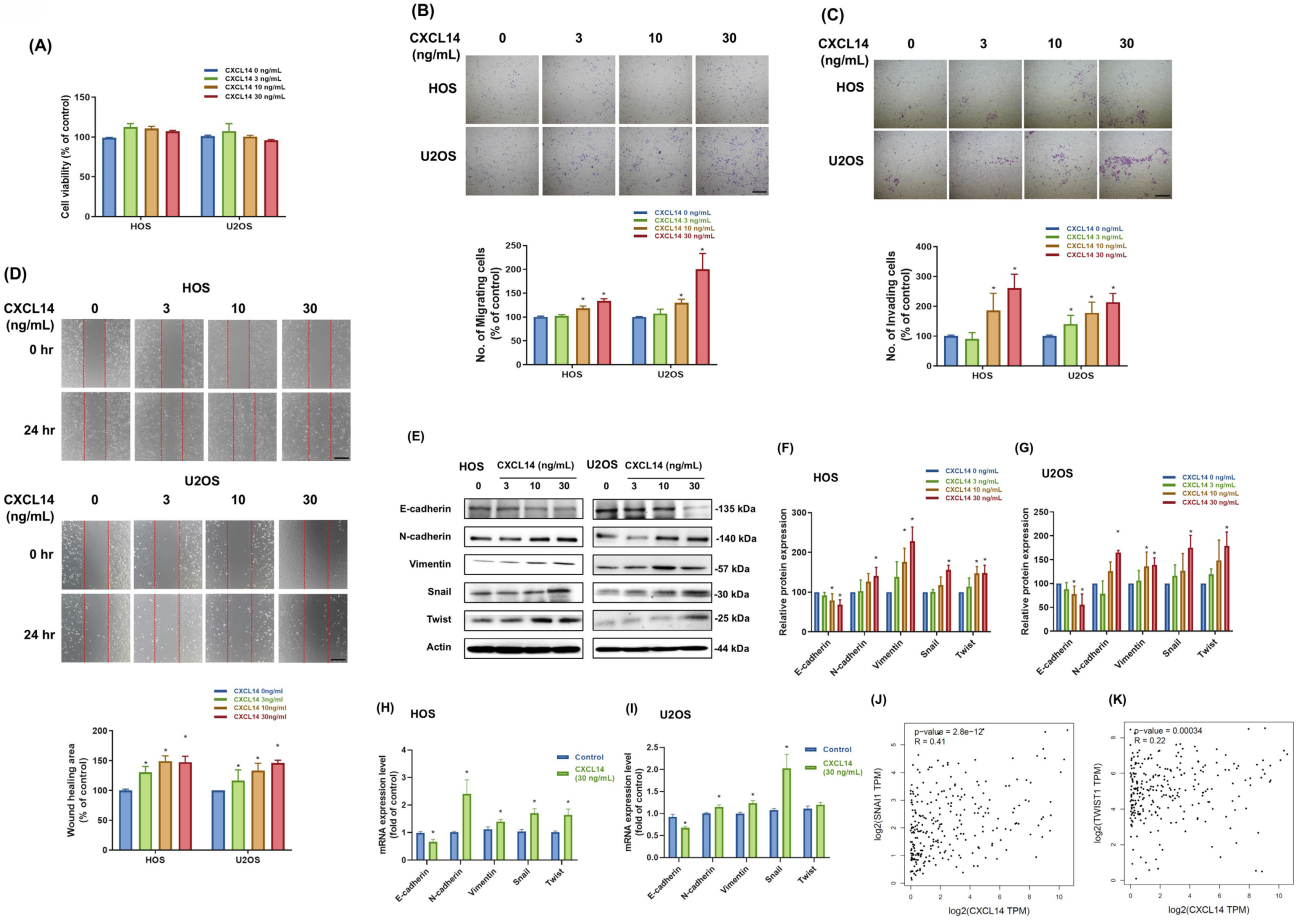
** CXCL14 promotes EMT in osteosarcoma cells.** (A) The cell viability of osteosarcoma cells (HOS and U2OS) in response to CXCL14 treatment (0, 3, 10, 30 ng/mL) was evaluated by cell viability assay. (B and C) HOS and U2OS osteosarcoma cells were treated with CXCL14 (0-30 ng/mL) for 24 hours. Subsequently, the Transwell assay was used to measure *in vitro* migration, and the Matrigel invasion assay was used to measure cell invasion. (D) HOS and U2OS osteosarcoma cells were incubated with CXCL14 (0-30 ng/mL) for 24 h, and cell migration was measured using the wound healing assay. (E) HOS and U2OS osteosarcoma cells were incubated with CXCL14 (0-30 ng/mL) for 24 h. Western Blot analysis was performed to examine the expression of E-cadherin, N-cadherin, vimentin, Snail, and Twist. β-actin was used as an internal control. (F and G) The quantification results of the Western blot are shown in Figure 3E. (H and I) HOS and U2OS osteosarcoma cells were incubated with CXCL14 (30 ng/mL) or vehicle control for 24 h. Total RNA was collected, and quantitative PCR (qPCR) analysis was used to determine the expression levels of *E-cadherin*, *N-cadherin*, *vimentin*, *Snail*, and *Twist*. (J and K) Correlation between CXCL14, Snail, and Twist expression levels in sarcoma specimens analyzed from the GEPIA online tool. Results are presented as the mean ± SEM from at least three independent experiments (N=3-6). *p < 0.05 compared to the control groups. Scale bar= 100 μm.

The activation of EMT can provide tumor cells with the ability to migrate 22. Thus, to investigate the role of CXCL14 in osteosarcoma EMT, we stimulated both HOS and U2OS cells with recombinant CXCL14. HOS cells possess a TP53 mutation and display high invasiveness, whereas U2OS cells retain the wild-type TP53 and are comparatively non-invasive 23-25. Subsequently, using these two genetically and phenotypically distinct lines, this study confirmed that CXCL14-induced EMT is not confined to a single aggressive background but is applicable across molecularly diverse osteosarcoma cell subtypes. Firstly, CXCL14 stimulation did not affect cell proliferation in these osteosarcoma cell lines (Fig. 3A). Hence, we further assessed the cell migratory ability using cell migration and wound healing assays. The results of these assays showed that CXCL14 stimulation promoted cell migration ability in osteosarcoma cells in a dose-dependent manner (Fig. 3B-3D). Next, the expression of EMT markers was investigated in osteosarcoma cells following CXCL14 treatment to confirm the induction of EMT.

Additional qPCR and Western blot analyses revealed the induction of EMT, indicated by a reduction in epithelial marker E-cadherin and an increase in mesenchymal markers, including N-cadherin, vimentin, Snail, and Twist (Fig. 3E-3I). Finally, we investigated the correlation between the expression levels of CXCL14, Snail, and Twist using GEPIA (Gene Expression Profiling Interactive Analysis)—a web-based platform that utilizes data from TCGA and the Genotype-Tissue Expression (GTEx) project 26. These results showed a positive correlation between CXCL14 expression and the expression of Twist and Snail in sarcoma samples (Fig. 3J and 3K), indicating that CXCL14 can promote EMT in osteosarcoma cells.

### AKT regulates CXCL14-promoted EMT in osteosarcoma cells

**Figure 4:**
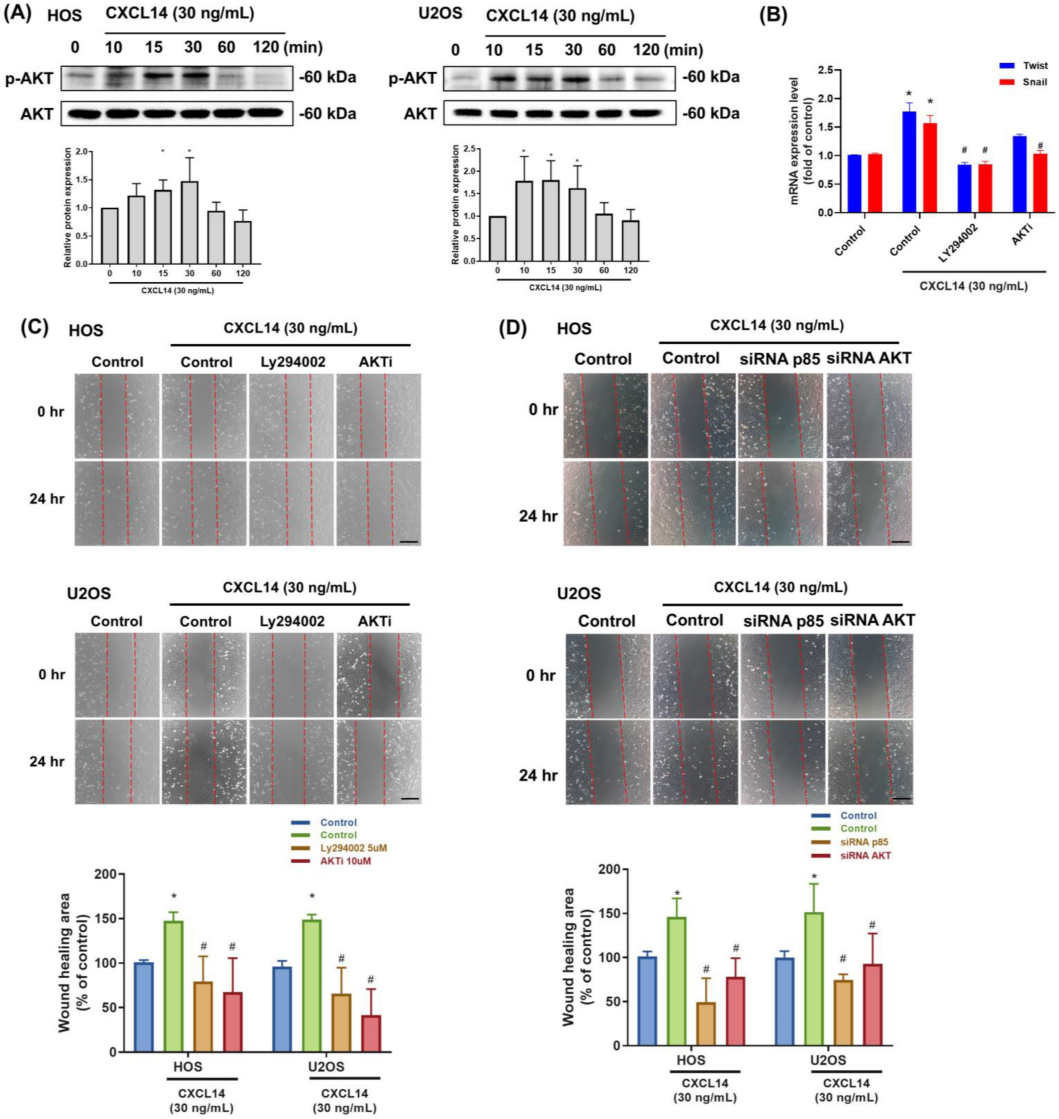
** CXCL14 promotes cell mobility in osteosarcoma through activation of the AKT signaling pathway.** (A) HOS and U2OS osteosarcoma cells were incubated with CXCL14 (30 ng/mL) for the indicated times, and AKT phosphorylation was determined by Western blot analysis. (B and C) HOS and U2OS osteosarcoma cells were pretreated with PI3K inhibitor (LY294002; 10 µM) or AKT inhibitor (AKTi; 10 µM) for 30 min, followed by stimulation with CXCL14 (30 ng/mL) for 24 h. Total RNA was extracted from HOS cells, and *Twist* and *Snail* mRNA levels were examined (B). Cell migration was evaluated by wound healing assay (C). (D) HOS and U2OS osteosarcoma cells were transfected with AKT siRNA for 24 hours and then incubated with CXCL14 (30 ng/mL) for an additional 24 hours. The wound healing assay was subsequently assessed. Results are presented as the mean ± SEM from at least three independent experiments (N=3-6). *p < 0.05 compared to the control group. #*p* < 0.05 compared to the CXCL14 treatment group. Scale bar= 100 μm.

The PI3K/Akt signaling pathway can impact EMT through multiple mechanisms, thereby influencing tumor aggressiveness 27. We first analyzed AKT activation in response to CXCL14 stimulation. These data revealed that CXCL14 increased phospho-AKT in osteosarcoma cells (Fig. 4A). To investigate the regulatory role of the PI3K/AKT signaling cascade in CXCL14-promoted EMT further, we evaluated the expression of Twist and Snail, two major transcriptional regulators involved in EMT induction 28, 29, following CXCL14 stimulation in the presence of PI3K or AKT inhibitors. Pretreatment with PI3K or AKT inhibitors (Ly294002 and AKTi) markedly suppressed the CXCL14-induced mRNA expression of *Twist* and *Snail* (Fig. 4B). Moreover, pretreatment with PI3K or AKT inhibitors significantly abolished cell migration after CXCL14 stimulation (Fig. 4C). Meanwhile, transfection with p85 or AKT siRNAs showed a similar response, in which CXCL14-promoted cell migration was inhibited (Fig. 4D). These data indicate that AKT participates in the induction of EMT in response to CXCL14 treatment in osteosarcoma cells.

### MAPK signaling pathways contribute to CXCL14-induced EMT in osteosarcoma cells

**Figure 5:**
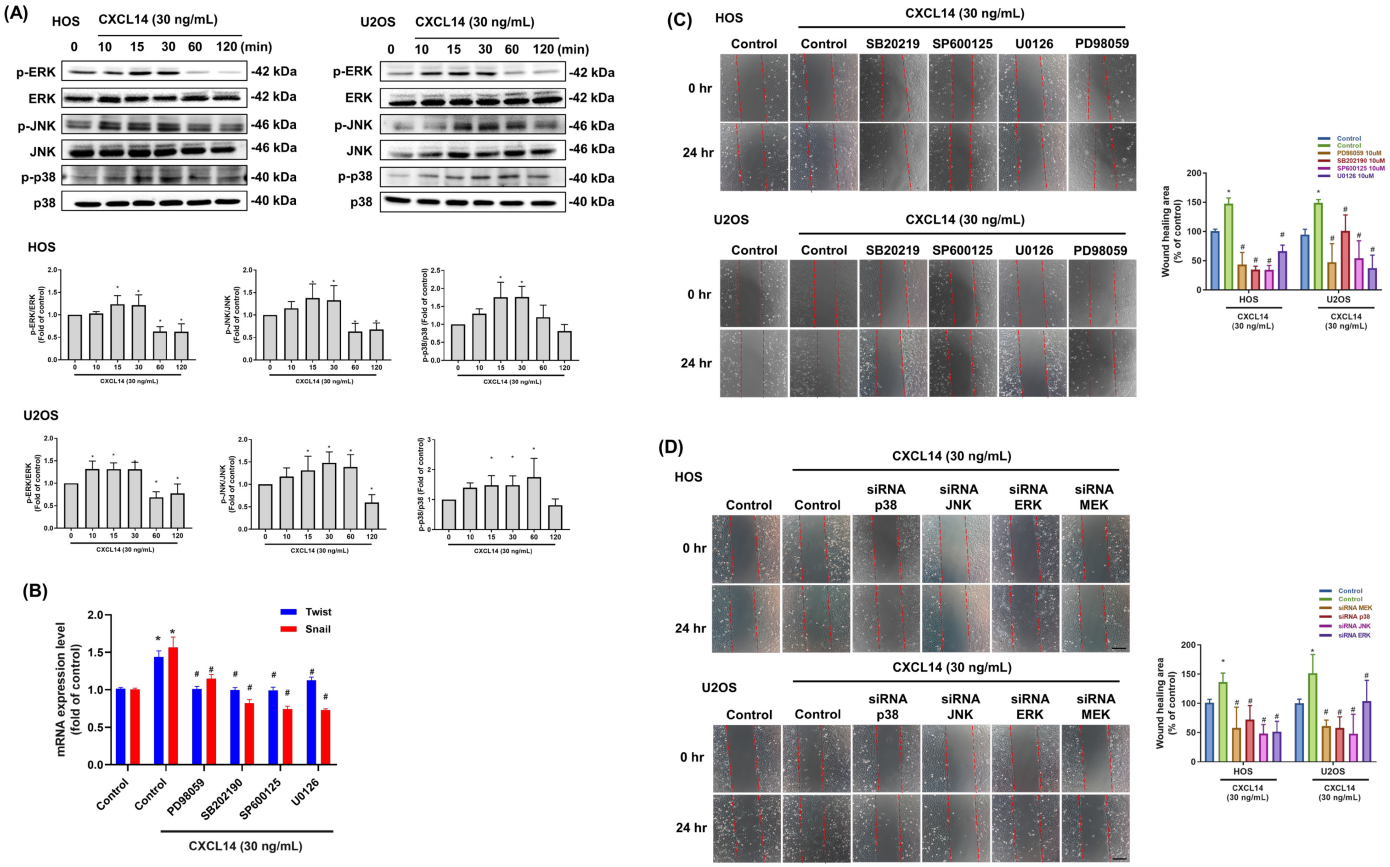
** CXCL14 promotes cell mobility in osteosarcoma through the MAPK signal pathway.** (A) HOS and U2OS osteosarcoma cells were incubated with CXCL14 (30 ng/mL) for the indicated times, and ERK, p38, and JNK phosphorylation were determined by Western Blot analysis. (B and C) HOS and U2OS osteosarcoma cells were pretreated with ERK inhibitor (PD98059, 10 μM; U0126, 10 μM), p38 inhibitor (SB202190, 10 μM), or JNK inhibitor (SP600125, 10 μM) for 30 minutes and then incubated with CXCL14 (30 ng/mL) for 24 hours. Total RNA was extracted from HOS cells, and the mRNA levels of *Twist* and *Snail* were examined (B). Cell migration was evaluated by wound healing assay (C). (D) HOS and U2OS osteosarcoma cells were transfected with ERK, p38, or JNK siRNAs for 24 hours and then incubated with CXCL14 (30 ng/mL) for an additional 24 hours. The wound healing assay was subsequently assessed. Results are presented as the mean ± SEM from at least three independent experiments (N=3-6). *p < 0.05 compared to the control group. #*p* < 0.05 compared to the CXCL14 treatment group. Scale bar= 100 μm.

A previous review demonstrated that the MAPK pathway is involved in the activation of EMT transcription factors 30. Moreover, MAPK cascades represent key elements in G protein-coupled receptor (GPCR)-induced intracellular signaling 31. Western blot analysis demonstrated that CXCL14 significantly activated the MAPK signaling pathway in osteosarcoma cells, as evidenced by the increased phosphorylation of key MAPK pathway proteins (ERK, JNK, and p38) (Fig. 5A). To elucidate the functional consequences of this activation further, we pretreated the osteosarcoma cells with specific MAPK pathway inhibitors. This pretreatment markedly reduced CXCL14-induced cell migration and the mRNA expression of *Twist* and *Snail*, indicating that the MAPK pathway plays a critical role in mediating the promigratory effects of CXCL14 (Fig. 5B and 5C). Additionally, to confirm the involvement of the MAPK pathway, we utilized siRNAs targeting essential components in this pathway. The knockdown of MAPK signaling using siRNA significantly decreased cell migration, indicating that CXCL14 promotes EMT and enhances cell migration in osteosarcoma cells primarily via MAPK pathway activation (Fig. 5D). These findings collectively highlight the MAPK signaling pathway as a key mediator of CXCL14-induced EMT.

### NF-κB transcription factor is required for CXCL14-promoted EMT in osteosarcoma cells

**Figure 6:**
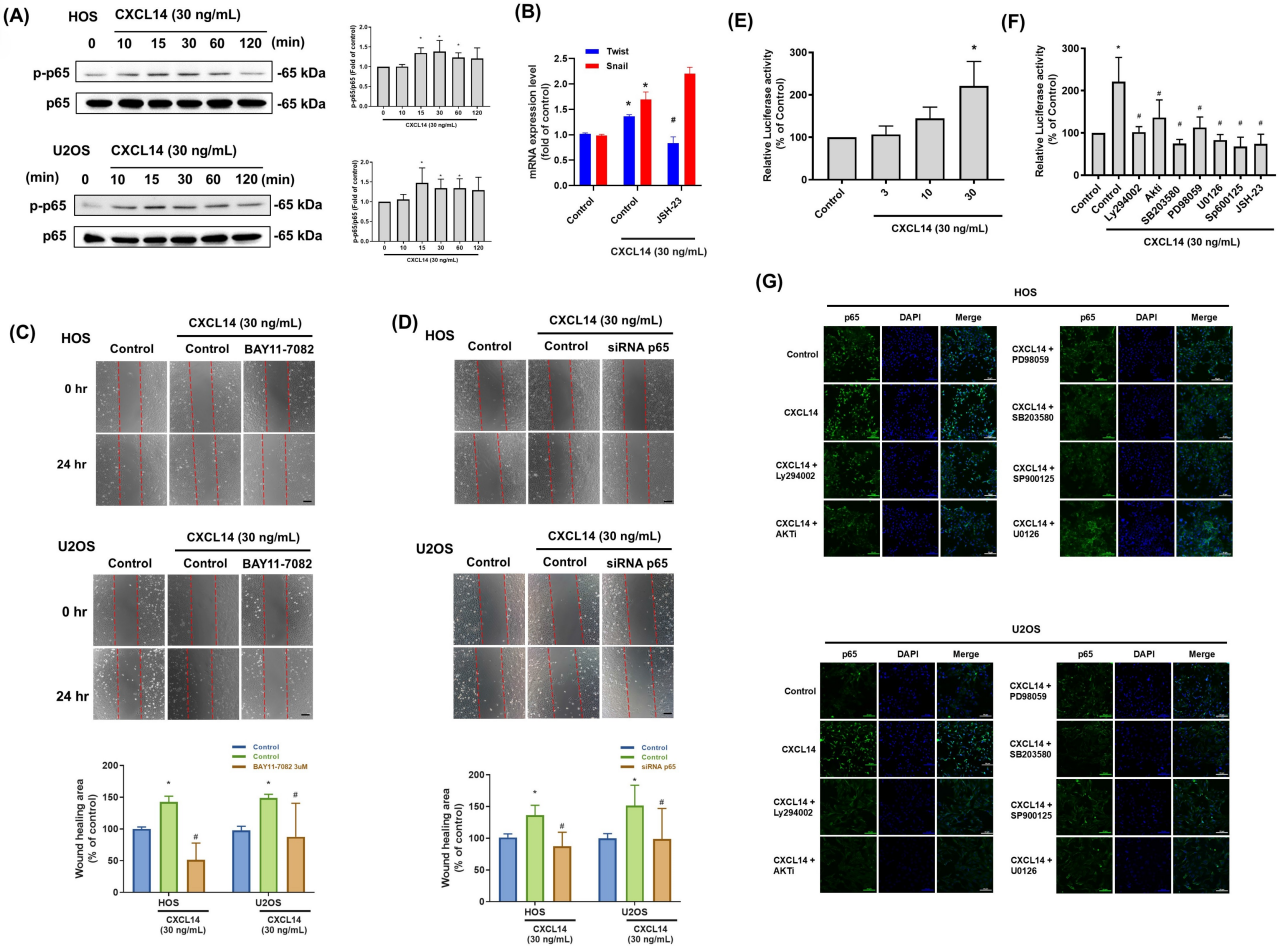
** CXCL14 promoted osteosarcoma cell migration through NF-κB activation.** (A) HOS and U2OS osteosarcoma cells were incubated with CXCL14 (30 ng/mL) for the indicated times, and p65 phosphorylation was determined by Western Blot analysis. (B and C) HOS and U2OS osteosarcoma cells were pretreated with an NF-κB inhibitor (BAY11-7082, 0.6 μM or JSH-23, 10 μM) for 30 minutes and then incubated with CXCL14 (30 ng/mL) for 24 hours. The wound healing assay was then performed. Total RNA was extracted from HOS cells, and the *Twist* and *Snail* mRNA levels were examined (B). Cell migration was evaluated by wound healing assay (C). (D) HOS and U2OS osteosarcoma cells were transfected with *p65* siRNA for 24 hours and then incubated with CXCL14 (30 ng/mL) for an additional 24 hours. The wound healing assay was subsequently performed. (E) NF-κB luciferase activity was measured in the HOS cells at various concentrations of CXCL14 stimulation. The results were normalized based on protein concentration. (F) The HOS cells were treated with CXCL14 (30 ng/mL) in the presence of different inhibitors (Ly294002, AKTi, U0126, PD98059, SB203580 (10 μM), SP600125, and JSH-23 (10 μM)), and NF-κB luciferase activity was measured and normalized according to the protein concentration. (G) The HOS and U2OS osteosarcoma cells were pretreated with various inhibitors (Ly294002, AKTi, U0126, PD98059, SB203580, SP600125) for 30 minutes, followed by incubation with CXCL14 (30 ng/mL) for 24 hours. Then, immunofluorescence staining was performed with anti-p65 antibody. Nuclei were counterstained with DAPI. The immunofluorescence staining results were photographed using a microscope. Results are presented as the mean ± SEM from at least three independent experiments (N=3-6). *p < 0.05 compared to the control group. #*p* < 0.05 compared to the CXCL14 treatment group. Scale bar= 50 μm.

GPCRs play a significant role in inflammation and cancer, with signaling cascades activated by GPCR ligands influencing NF-κB activity either directly, by modifying upstream signaling events, or indirectly, through activating other transcription factors that bind to promoter regions adjacent to NF-κB 32. CXCL14 treatment increased p65 phosphorylation in osteosarcoma cells (Fig. 6A). Furthermore, inhibition of NF-κB activity using the inhibitors JSH-23, BAY11-7082, or p65 siRNA strongly suppressed CXCL14-induced cell migration and the mRNA expression of *Twist* and *Snail* in osteosarcoma cells (Fig. 6B-6D). NF-κB activation in response to CXCL14 stimulation was also confirmed by luciferase reporter assay (Fig. 6E). The PI3K, AKT, JNK, ERK, p38, and p65 inhibitors drastically reduced the CXCL14-regulated NF-κB activation in the osteosarcoma cells (Fig. 6F). Finally, immunofluorescence staining was conducted to monitor p65 nuclear translocation. These results revealed that CXCL14 promoted the nuclear translocation of p65, whereas pretreatment with the PI3K, AKT, JNK, ERK, and p38 inhibitors abrogated this phenomenon (Fig. 6G). In summary, these data prove that NF-κB activation is required for CXCL14-induced EMT in osteosarcoma cells.

### CXCL14 knockdown reduced tumor growth in an orthotopic animal model

**Figure 7:**
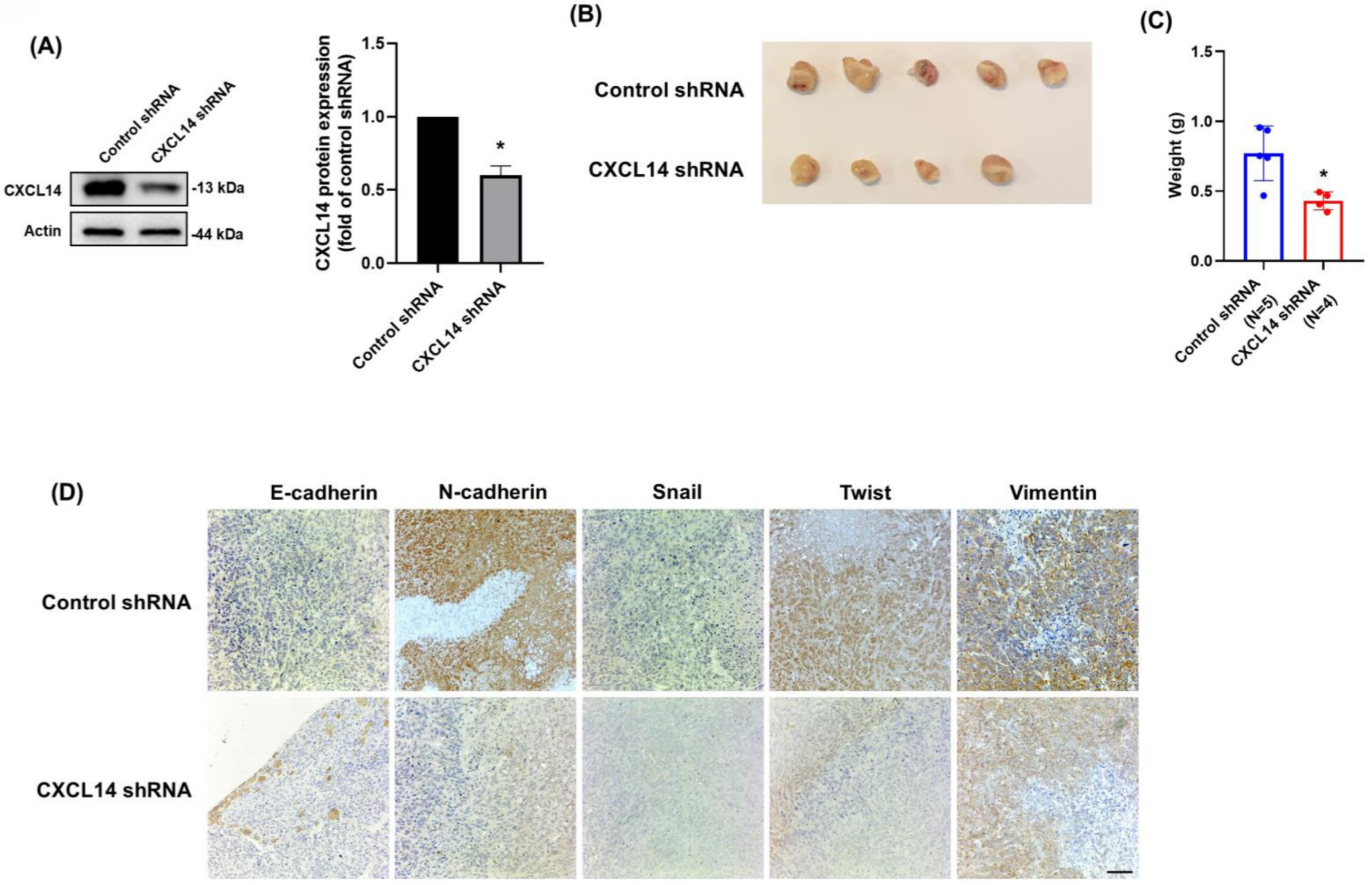
** CXCL14 knockdown inhibited osteosarcoma tumor cell growth.** (A) Total protein was collected from 143B cells stably expressing shRNAs directed against *CXCL14* or control shRNA. Western blot analysis was used to detect CXCL14. β-actin was used as the loading control. (B) The tumors that grew in the tibias of the mice were collected and photographed. (C) Quantification of tumor weights following intratibial injection. (D) The tumors were collected from mice that received intratibial injections of 143B control shRNA and CXCL14 shRNA cells. Immunohistochemistry staining was performed to evaluate the expression of E-cadherin, N-cadherin, Snail, Twist, and vimentin. Results are presented as the mean ± SEM from at least three independent experiments (N=3-6). *p < 0.05 compared to the control shRNA group. Scale bar= 50 μm.

The *in vitro* findings demonstrated that CXCL14 promoted osteosarcoma cell migration by inducing EMT. Therefore, we utilized 143B osteosarcoma cells for an animal study, as these cells are aggressive and readily grow on the tibia of mice. The CXCL14 expression level was confirmed in CXCL14 stable knockdown and control shRNA cell lines (Fig. 7A). The orthotopic xenograft animal model was conducted through an intratibial injection of 143B stable clones. Six weeks after tumor implantation, the mice were sacrificed, and tumors were photographed and weighed. The results showed that CXCL14 knockdown greatly inhibited bone tumor growth (Fig. 7B and 7C). Furthermore, the EMT markers, including E-cadherin, N-cadherin, Snail, Twist, and vimentin, were assessed in tumor specimens. The results were in accordance with the *in vitro* findings that CXCL14 knockdown could prevent EMT in osteosarcoma cells (Fig. 7D). This evidence suggested that CXCL14 promotes tumor growth in osteosarcoma *in vivo*.

## Discussion

CXCL14, a chemokine known for its roles in immune cell recruitment and tumor biology, exhibits a highly variable expression pattern across different tumors. This variability reflects its complex and context-dependent functions in cancer progression, including both tumor-suppressive and tumor-promoting effects, as well as modulation of the tumor microenvironment 33. Notably, CXCL14 can function as a tumor suppressor in certain malignancies. For example, CXCL14 is frequently downregulated in human papillomavirus (HPV)-associated head and neck squamous cell carcinoma (HNSCC) and cervical cancer, a loss that is thought to help tumors evade immune surveillance 34, 35. Meanwhile, the restoration of CXCL14 expression in HPV-positive cancer cells has been shown to recruit natural killer and T cells, and significantly suppress tumor growth *in vivo* through enhanced antitumor immunity 35. Similarly, the *CXCL14* gene is often silenced in lung adenocarcinoma through promoter hypermethylation, and the enforced re-expression of *CXCL14* in these tumor models induces cancer cell necrosis and impairs tumor progression 36. These findings suggest that CXCL14 can act as a context-dependent tumor suppressor, enhancing antitumor immunity and directly inhibiting tumor cell viability.

In contrast, CXCL14 is upregulated in several tumor types, where it plays a significant role in promoting tumor progression. In breast cancer, particularly in the aggressive triple-negative subtype (TNBC), CXCL14 contributes to tumor progression by promoting EMT 12 and enhancing the invasive and metastatic behavior of cancer cells 37. Similarly, elevated CXCL14 levels are associated with advanced disease stages in prostate cancer 38 and poor prognosis lung cancer 39, 40. Previous research has demonstrated that CXCL14 is overexpressed in osteosarcoma and is associated with a poor prognosis 19. Additionally, the upregulation of CXCL14 in prostate-derived cancer-associated fibroblasts (CAFs) suggests that CXCL14 expression represents a key marker of metastatic prostate cancer in humans 41. Our findings are in accordance with previous evidence in osteosarcoma cells, indicating that CXCL14 expression may enhance the malignancy of osteosarcoma. Hence, these contrasting observations collectively emphasize the dual role of CXCL14 in cancer, acting either as a tumor suppressor or promoter depending on cellular context and tumor microenvironment. Recent single-cell analyses have identified CXCL14 enrichment in stem-like osteosarcoma subpopulations 42. Consistent with the well-established link between EMT and cancer stemness, our highly metastatic M10 subclone characterized by elevated Snail and Twist—likely represents a population with acquired stem-like traits. This suggests that CXCL14 signaling confers not only migratory potential but also stemness properties that fuel aggressive tumor progression.

This study found that HOS cells exhibit a more potent invasive response to CXCL14 compared to U2OS cells. This finding is directly consistent with previous research, which has established that HOS cells possess superior intrinsic invasive potential 25. Therefore, our results suggest that CXCL14 functions as a key signaling molecule that amplifies the pre-existing, aggressive phenotype of HOS cells. Furthermore, CXCL14 was found to promote EMT and contribute to the malignancy of osteosarcoma cells, which is in agreement with previous reports in breast cancer 12 and lung cancer 43. CXCL14 secreted by CAFs reduces epithelial markers, such as E-cadherin and cytokeratins 8 and 18, while enhancing mesenchymal markers, including vimentin, α-smooth muscle actin, and matrix metalloproteinase-2 via ERK1/2 signaling. This pathway activation increases EMT-related transcription factors, such as Slug and Twist 12. Although the specific receptor mediating CXCL14 signaling in our osteosarcoma model was not definitively identified in this study, recent literature has provided significant insights into its receptor interactions in the context of metastasis. Notably, a recent study identified Integrin α11β1 on cancer-associated fibroblasts as a key receptor for CXCL14, which facilitates the formation of a lung metastatic niche in osteosarcoma 42. A previous study also explored the role of chemokine ligand 14 (CXCL14) and its receptor, atypical chemokine receptor 2 (ACKR2), in promoting EMT and metastasis in lung cancer 43. This study identified that CXCL14 suppresses E-cadherin while upregulating N-cadherin, vimentin, and Snail expression. These findings suggest that CXCL14 influences the aggressiveness of cancer cells by regulating the EMT process. Furthermore, CXCL14 is implicated in the processes of immune cell infiltration, dendritic cell maturation, the upregulation of major histocompatibility complex I, and cellular mobilization 33. The expression of chemokines significantly affects the localization of immune cells within the tumor microenvironment and modulates the immune response directed against neoplastic cells, thereby playing a crucial role in cancer progression and metastasis 44. It is proposed that CXCL14 expression contributes to the heterogeneity observed in immune cell activation and infiltration, which may facilitate antitumor effects by enhancing immune responses and suppressing tumor growth, as evidenced in gastrointestinal stromal tumors 45, cervical cancer 46, and renal cancer 47. This discrepancy underscores the tissue-specific and context-dependent nature of CXCL14 in oncology—a 'double-edged sword.' In cancers where CXCL14 is associated with favorable outcomes, its function is largely attributed to the recruitment of cytotoxic immune cells (NK and T cells) into the tumor microenvironment. However, in the context of osteosarcoma, our findings suggest that the tumor-intrinsic activation of EMT and PI3K/MAPK signaling may dominate over these immune-mediated benefits, ultimately driving metastasis and poor patient survival. This highlights the importance of targeting the specific downstream effectors of CXCL14 in osteosarcoma therapeutics. However, the cancer/testis antigens NY-ESO-1 and MAGE-A4 are highly expressed in approximately 40-50% of osteosarcomas and are already being explored in peptide-, T cell receptor-, and chimeric antigen receptor-based immunotherapy trials 48, 49. Combining CXCL14-targeted strategies (to curb invasion and metastasis) with NY-ESO-1/MAGE-A4-directed immunotherapies (to enhance tumor-specific cytotoxicity) could therefore represent a novel, complementary approach for osteosarcoma treatment.

Beyond promoting cancer cell proliferation, MAPK signaling is also involved in regulating tumor metastasis 50. Augsten *et al*. proposed that CAFs mediate the autocrine secretion of CXCL14 within the prostate cancer microenvironment. Meanwhile, increased CXCL14 expression in NIH-3T3 murine fibroblasts was shown to promote fibroblast proliferation and migration through ERK1/2 signaling 51. Furthermore, a previous study demonstrated that p38/JNK are required for CXCL14-promoted cell migration and EMT in lung cancer cells 43. Our findings suggest a pivotal role for MAPK in CXCL14-promoted tumor progression. To provide a comprehensive view of the metastatic hierarchy in osteosarcoma, it is essential to link our downstream findings with the upstream regulators of CXCL14. Previous studies have established that hypomethylation of the IRX1 promoter is a primary epigenetic driver leading to CXCL14 overexpression in osteosarcoma 52. CXCL14 stimulates the proliferation and migration of lung cancer cells that overexpress glycoproteins containing heparan sulfate and sialic acid, an effect mediated through NF-κB activation 53. Elevated CXCL14 expression in osteosarcoma cell lines triggered the NF-κB activation, resulting in increased levels of the anti-apoptotic genes *c-FLIP* and *MMP9*. This process inhibited anoikis and facilitated osteosarcoma metastasis 54. Our current study aligns with previous works, revealing that NF-κB plays a crucial role in CXCL14-driven metastasis.

## Conclusion

The present study explored the mechanisms through which CXCL14 promotes EMT in osteosarcoma and tumor growth *in vivo*, a process that remains unclear based on our review of the existing literature. Our findings indicate that PI3K/AKT, MAPK, and NF-κB are sequentially involved in the EMT and migration of osteosarcoma cells. This signaling pathway could shed light on the mechanisms of osteosarcoma metastasis and potentially guide the development of more effective therapies in the future.

## Data Availability

The data sets used and analyzed during the current study are available from the corresponding author on reasonable request.

## References

[1] Organisation mondiale de la s, Centre international de recherche sur le c WHO classification of tumours of soft tissue and bone. Lyon: International agency for research on cancer (IARC) Press.

[2] Mohseny AB, Szuhai K, Romeo S, Buddingh EP, Briaire-de Bruijn I, de Jong D (2009). Osteosarcoma originates from mesenchymal stem cells in consequence of aneuploidization and genomic loss of Cdkn2. J Pathol.

[3] Wang SW, Wu HH, Liu SC, Wang PC, Ou WC, Chou WY (2012). CCL5 and CCR5 interaction promotes cell motility in human osteosarcoma. PLoS One.

[4] Harting MT, Blakely ML (2006). Management of osteosarcoma pulmonary metastases. Semin Pediatr Surg.

[5] PosthumaDeBoer J, Witlox MA, Kaspers GJ, van Royen BJ (2011). Molecular alterations as target for therapy in metastatic osteosarcoma: a review of literature. Clin Exp Metastasis.

[6] Thiery JP, Acloque H, Huang RY, Nieto MA (2009). Epithelial-mesenchymal transitions in development and disease. Cell.

[7] Thiery JP (2002). Epithelial-mesenchymal transitions in tumour progression. Nat Rev Cancer.

[8] Thompson EW, Newgreen DF, Tarin D (2005). Carcinoma invasion and metastasis: a role for epithelial-mesenchymal transition?. Cancer Res.

[9] Yang G, Yuan J, Li K (2013). EMT transcription factors: implication in osteosarcoma. Med Oncol.

[10] Miller MC, Mayo KH (2017). Chemokines from a Structural Perspective. International journal of molecular sciences.

[11] Frederick MJ, Henderson Y, Xu X, Deavers MT, Sahin AA, Wu H (2000). In vivo expression of the novel CXC chemokine BRAK in normal and cancerous human tissue. The American journal of pathology.

[12] Sjoberg E, Meyrath M, Milde L, Herrera M, Lovrot J, Hagerstrand D (2019). A Novel ACKR2-Dependent Role of Fibroblast-Derived CXCL14 in Epithelial-to-Mesenchymal Transition and Metastasis of Breast Cancer. Clin Cancer Res.

[13] Eiro N, Fernandez-Gomez J, Sacristan R, Fernandez-Garcia B, Lobo B, Gonzalez-Suarez J (2017). Stromal factors involved in human prostate cancer development, progression and castration resistance. Journal of cancer research and clinical oncology.

[14] Liu Y, Zhang J, Sun X, Su Q, You C (2017). Down-regulation of miR-29b in carcinoma associated fibroblasts promotes cell growth and metastasis of breast cancer. Oncotarget.

[15] Ozawa S, Kato Y, Kubota E, Hata R (2009). BRAK/CXCL14 expression in oral carcinoma cells completely suppresses tumor cell xenografts in SCID mouse. Biomedical research.

[16] Hata R, Izukuri K, Kato Y, Sasaki S, Mukaida N, Maehata Y (2015). Suppressed rate of carcinogenesis and decreases in tumour volume and lung metastasis in CXCL14/BRAK transgenic mice. Scientific reports.

[17] Lin Y, Chen BM, Yu XL, Yi HC, Niu JJ, Li SL (2019). Suppressed Expression of CXCL14 in Hepatocellular Carcinoma Tissues and Its Reduction in the Advanced Stage of Chronic HBV Infection. Cancer management and research.

[18] Hara T, Tanegashima K (2012). Pleiotropic functions of the CXC-type chemokine CXCL14 in mammals. J Biochem.

[19] Liang W, Yang C, Peng J, Qian Y, Wang Z (2015). The Expression of HSPD1, SCUBE3, CXCL14 and Its Relations with the Prognosis in Osteosarcoma. Cell Biochem Biophys.

[20] Li WH, Wu HJ, Li YX, Pan HG, Meng T, Wang X (2016). MicroRNA-143 promotes apoptosis of osteosarcoma cells by caspase-3 activation via targeting Bcl-2. Biomed Pharmacother.

[21] Liu JF, Chen PC, Chang TM, Hou CH (2020). Monocyte Chemoattractant Protein-1 promotes cancer cell migration via c-Raf/MAPK/AP-1 pathway and MMP-9 production in osteosarcoma. J Exp Clin Cancer Res.

[22] Yang J, Antin P, Berx G, Blanpain C, Brabletz T, Bronner M (2020). Guidelines and definitions for research on epithelial-mesenchymal transition. Nat Rev Mol Cell Biol.

[23] Osaki M, Takeshita F, Sugimoto Y, Kosaka N, Yamamoto Y, Yoshioka Y (2011). MicroRNA-143 regulates human osteosarcoma metastasis by regulating matrix metalloprotease-13 expression. Mol Ther.

[24] Yuan TL, Fellmann C, Lee CS, Ritchie CD, Thapar V, Lee LC (2014). Development of siRNA payloads to target KRAS-mutant cancer. Cancer Discov.

[25] Lauvrak SU, Munthe E, Kresse SH, Stratford EW, Namlos HM, Meza-Zepeda LA (2013). Functional characterisation of osteosarcoma cell lines and identification of mRNAs and miRNAs associated with aggressive cancer phenotypes. Br J Cancer.

[26] Chen P-C, Tang C-H, Lin L-W, Tsai C-H, Chu C-Y, Lin T-H (2017). Thrombospondin-2 promotes prostate cancer bone metastasis by the up-regulation of matrix metalloproteinase-2 through down-regulating miR-376c expression. Journal of hematology & oncology.

[27] Xu W, Yang Z, Lu N (2015). A new role for the PI3K/Akt signaling pathway in the epithelial-mesenchymal transition. Cell Adh Migr.

[28] Kaufhold S, Bonavida B (2014). Central role of Snail1 in the regulation of EMT and resistance in cancer: a target for therapeutic intervention. J Exp Clin Cancer Res.

[29] Huang Y, Hong W, Wei X (2022). The molecular mechanisms and therapeutic strategies of EMT in tumor progression and metastasis. J Hematol Oncol.

[30] Sabbah M, Emami S, Redeuilh G, Julien S, Prevost G, Zimber A (2008). Molecular signature and therapeutic perspective of the epithelial-to-mesenchymal transitions in epithelial cancers. Drug Resist Updat.

[31] Naor Z, Benard O, Seger R (2000). Activation of MAPK cascades by G-protein-coupled receptors: the case of gonadotropin-releasing hormone receptor. Trends Endocrinol Metab.

[32] Fraser CC (2008). G protein-coupled receptor connectivity to NF-kappaB in inflammation and cancer. Int Rev Immunol.

[33] Gowhari Shabgah A, Haleem Al-Qaim Z, Markov A, Valerievich Yumashev A, Ezzatifar F, Ahmadi M (2021). Chemokine CXCL14; a double-edged sword in cancer development. Int Immunopharmacol.

[34] Cicchini L, Blumhagen RZ, Westrich JA, Myers ME, Warren CJ, Siska C (2017). High-Risk Human Papillomavirus E7 Alters Host DNA Methylome and Represses HLA-E Expression in Human Keratinocytes. Sci Rep.

[35] Westrich JA, Vermeer DW, Silva A, Bonney S, Berger JN, Cicchini L (2019). CXCL14 suppresses human papillomavirus-associated head and neck cancer through antigen-specific CD8(+) T-cell responses by upregulating MHC-I expression. Oncogene.

[36] Tessema M, Klinge DM, Yingling CM, Do K, Van Neste L, Belinsky SA (2010). Re-expression of CXCL14, a common target for epigenetic silencing in lung cancer, induces tumor necrosis. Oncogene.

[37] Pelicano H, Lu W, Zhou Y, Zhang W, Chen Z, Hu Y (2009). Mitochondrial dysfunction and reactive oxygen species imbalance promote breast cancer cell motility through a CXCL14-mediated mechanism. Cancer Res.

[38] Williams KA, Lee M, Hu Y, Andreas J, Patel SJ, Zhang S (2014). A systems genetics approach identifies CXCL14, ITGAX, and LPCAT2 as novel aggressive prostate cancer susceptibility genes. PLoS Genet.

[39] Sata Y, Nakajima T, Fukuyo M, Matsusaka K, Hata A, Morimoto J (2020). High expression of CXCL14 is a biomarker of lung adenocarcinoma with micropapillary pattern. Cancer Sci.

[40] Ji X, Shen Z, Zhao B, Yuan X, Zhu X (2018). CXCL14 and NOS1 expression in specimens from patients with stage I-IIIA nonsmall cell lung cancer after curative resection. Medicine (Baltimore).

[41] Song EY, Shurin MR, Tourkova IL, Gutkin DW, Shurin GV (2010). Epigenetic mechanisms of promigratory chemokine CXCL14 regulation in human prostate cancer cells. Cancer Res.

[42] Xu Y, Deng C, Chen H, Song Y, Xu H, Song G (2024). Osteosarcoma Cells Secrete CXCL14 That Activates Integrin α11β1 on Fibroblasts to Form a Lung Metastatic Niche. Cancer Res.

[43] Chang TM, Chiang YC, Lee CW, Lin CM, Fang ML, Chi MC (2023). CXCL14 promotes metastasis of non-small cell lung cancer through ACKR2-depended signaling pathway. Int J Biol Sci.

[44] Marcuzzi E, Angioni R, Molon B, Cali B (2018). Chemokines and Chemokine Receptors: Orchestrating Tumor Metastasization. Int J Mol Sci.

[45] Vitiello GA, Bowler TG, Liu M, Medina BD, Zhang JQ, Param NJ (2019). Differential immune profiles distinguish the mutational subtypes of gastrointestinal stromal tumor. J Clin Invest.

[46] Cicchini L, Westrich JA, Xu T, Vermeer DW, Berger JN, Clambey ET (2016). Suppression of Antitumor Immune Responses by Human Papillomavirus through Epigenetic Downregulation of CXCL14. mBio.

[47] Lyu XJ, Li HZ, Ma X, Li XT, Gao Y, Ni D (2015). Elevated S100A6 (Calcyclin) enhances tumorigenesis and suppresses CXCL14-induced apoptosis in clear cell renal cell carcinoma. Oncotarget.

[48] Hashimoto K, Nishimura S, Ito T, Oka N, Kakinoki R, Akagi M (2022). Clinicopathological assessment of cancer/testis antigens NY-ESO-1 and MAGE-A4 in osteosarcoma. Eur J Histochem.

[49] Ren S, Zhang Z, Li M, Wang D, Guo R, Fang X (2023). Cancer testis antigen subfamilies: Attractive targets for therapeutic vaccine (Review). Int J Oncol.

[50] Kciuk M, Gielecinska A, Budzinska A, Mojzych M, Kontek R (2022). Metastasis and MAPK Pathways. Int J Mol Sci.

[51] Augsten M, Hagglof C, Olsson E, Stolz C, Tsagozis P, Levchenko T (2009). CXCL14 is an autocrine growth factor for fibroblasts and acts as a multi-modal stimulator of prostate tumor growth. Proc Natl Acad Sci U S A.

[52] Lu J, Song G, Tang Q, Zou C, Han F, Zhao Z (2015). IRX1 hypomethylation promotes osteosarcoma metastasis via induction of CXCL14/NF-κB signaling. J Clin Invest.

[53] Park CR, You DJ, Kim DK, Moon MJ, Lee C, Oh SH (2013). CXCL14 enhances proliferation and migration of NCI-H460 human lung cancer cells overexpressing the glycoproteins containing heparan sulfate or sialic acid. J Cell Biochem.

[54] Lu J, Song G, Tang Q, Zou C, Han F, Zhao Z (2015). IRX1 hypomethylation promotes osteosarcoma metastasis via induction of CXCL14/NF-kappaB signaling. J Clin Invest.

